# Assessment of Oxidant and Antioxidant Status in Prepubertal Children following Vegetarian and Omnivorous Diets

**DOI:** 10.3390/antiox12030682

**Published:** 2023-03-10

**Authors:** Grażyna Rowicka, Witold Klemarczyk, Jadwiga Ambroszkiewicz, Małgorzata Strucińska, Ewa Kawiak-Jawor, Halina Weker, Magdalena Chełchowska

**Affiliations:** 1 Department of Nutrition, Institute of Mother and Child, 01-211 Warsaw, Poland; 2 Department of Screening Tests and Metabolic Diagnostics, Institute of Mother and Child, 01-211 Warsaw, Poland; 3 Łukasiewicz Research Network, Institute of Organization and Management in Industry “ORGMASZ”, 00-879 Warsaw, Poland

**Keywords:** lacto-ovo-vegetarian diet, children, reduced glutathione, oxidized glutathione, total oxidant capacity, total antioxidant capacity

## Abstract

Oxidant-antioxidant balance is crucial for maintaining one’s health, and the diet is possibly one of the most important factors affecting this balance. Therefore, the aim of this study was to determine the oxidant-antioxidant balance in children on a lacto-ovo-vegetarian diet. The study was conducted between January 2020 and December 2021. The concentrations of total oxidant capacity (TOC), total antioxidant capacity (TAC), reduced (GSH), and oxidized (GSSG) glutathione, as well as C-reactive protein (CRP) and calprotectin were measured in serum samples of 72 healthy prepubertal children (32 vegetarians and 40 omnivores). The oxidative stress index (OSI) and the GSH/GSSG ratio (R-index) were calculated. Children on a vegetarian diet had significantly lower median values of TOC, GSH, and GSSG, and higher TAC compared with the omnivores. OSI was significantly lower in vegetarians, while R-index, as well as median values of CRP and calprotectin did not differ between both groups of children. Significant negative correlations were observed between TOC and TAC levels in the whole group of children and in vegetarians. GSH and GSSG levels correlated positively in the groups of vegetarians, omnivores, and in all the children. There were significant positive correlations between TOC and GSH, as well as GSSG levels in all the studied groups of children. Our study results suggest that the vegetarian model of nutrition allows to maintain the oxidant-antioxidant balance in the serum of prepubertal children.

## 1. Introduction

In recent years, there has been growing interest in unconventional diets, including the vegetarian model of nutrition. According to the available data (consumer research), the number of adults in Poland who prefer a vegetarian diet amounts to approximately 8%, while the percentage of children eating unconventionally (i.e., whose parents consciously eliminate certain foods from their diet not for health reasons) is estimated at 0.5–4.5%. The variation of the vegetarian diet considered to be one of the least restrictive is lacto-ovo-vegetarianism. Lacto-ovo-vegetarians do not consume meat and fish, as well as foods that contain them, but their diets include eggs, milk, and dairy products. According to the current knowledge, children can be on a vegetarian diet, provided that it is properly balanced [1]. However, some authors emphasize that it is difficult to properly balance a vegetarian diet, especially in infants during the period of expanding their diet, which may adversely affect their physical and neurocognitive development [2]. Therefore, some scientific societies recommend that in infants and young children whose parents decide to feed them a vegetarian diet, it should be a lacto-ovo-vegetarian diet [3,4,5].

Despite the documented health benefits of a vegetarian diet, which is most extensively studied in adults, attention is drawn to the fact that long-term use of any type of unbalanced diet, including a vegetarian diet, may lead to nutritional deficiencies [6,7,8,9,10].

In vegetarian diets, this applies primarily to selected exogenous amino acids, long-chain polyunsaturated fatty acids (PUFAs), minerals such as iron, calcium, and zinc, and vitamins, mainly B_12_ and D [10,11,12,13,14]. Possible consequences of deficiencies—particularly of those ingredients that have anti-inflammatory and antioxidant properties—include not only the induction of low-grade inflammation, but also excessive reactive oxygen species (ROS) activity. ROS are natural products of aerobic cellular metabolism and in physiological concentrations play an important role in the appropriate functioning of complex mechanisms, which control cell division and participate in many important cellular processes. Such processes include, inter alia, the activation of transcription factors, such as Nrf2 (nuclear factor-erythroid 2-related factor 2) or NF-kB (kappa-light-chain-enhancer of activated B cells), the regulation of protein phosphorylation processes, or the level of calcium in cells, etc. They are also defense agents of the body, participating in the elimination of microorganisms in the process of phagocytosis. Activation of the NF-κB pathway triggers the transcription of many genes involved in further stages of the response, including in inducing inflammation and stimulating cell proliferation. It is also a key regulator of inflammatory processes, immune response, and apoptosis. Apart from these important biological functions, ROS may also be agents damaging the cellular components [15,16]. In in vitro conditions, free radicals cause modifications and damage lipids, proteins, carbohydrates, and nucleotides, inducing changes in the DNA structure leading to mutations or cytotoxic effects. Free radicals formed in the body are eliminated through antioxidant mechanisms, including enzymes, e.g., superoxide dismutase (Cu/ZnSOD), catalase (CAT), glutathione peroxidase (GPx), glutathione reductase (GR), and glutathione transferases (GST), and small molecule antioxidants of both endogenous and exogenous origins [17]. Among endogenous low-molecular antioxidants, glutathione plays a particularly important role. It is an intracellular peptide belonging to the thiol group, i.e., chemical compounds containing sulfur, composed of three amino acids—glutamic acid, cysteine, and glycine. Key determinants of GSH synthesis, apart from the availability of the sulfur amino acid precursor, cysteine, include the activity of the enzyme, glutamate cysteine ligase (GCL), which is composed of a catalytic (GCLC) and a modifier (GCLM) subunit, and of the GSH synthetase (GS) enzyme [18]. Its reducing properties rely on an interaction with hydrogen peroxide and organic peroxides. Simultaneously, it enables the regeneration of antioxidant vitamins, e.g., ascorbic acid, α-tocopherol, and maintaining the thiol groups of proteins in a reduced state. It is one of the key factors affecting the oxidoreductive status in cells. Exogenous low-molecular antioxidants include vitamin A, β-carotene, vitamins C, E, B_12,_ and D, the serum concentrations of which, except for vitamin D, depend mainly on the amount of intake of these compounds with food [17,19,20,21,22,23].

There are few studies assessing the oxidant and antioxidant status in children on a lacto-ovo-vegetarian diet, but they seem to be particularly important due to the fact that imbalances between pro-oxidants and antioxidants leading to oxidative stress during metabolic programming, apart from consequences in the present, may also have long-term adverse health effects [24]. 

The aim of the study was to evaluate the potential severity of oxidative processes and antioxidant defense capacity in children on a lacto-ovo-vegetarian diet in relation to omnivorous children. Therefore, the serum concentrations of total oxidative capacity (TOC), total antioxidant capacity (TAC), reduced (GSH), and oxidized (GSSG) forms of glutathione in vegetarian and omnivorous children were assessed.

## 2. Materials and Methods

The protocol of this study was in accordance with the Helsinki Declaration of Principles and approved by the Ethics Committee of the Institute of Mother and Child, Warsaw, Poland (decision number 12/2019, 12 March 2019). All children’s parents were informed about the study procedures and all signed a written consent prior to the start of the study. 

### 2.1. Subjects

The study was conducted at the Institute of Mother and Child in Warsaw among patients of the Gastroenterology Outpatient Clinic between January 2020 and December 2021. 

We examined 72 healthy prepubertal Caucasian children aged 2–10 years. Among them, there were 32 children (47% male, 53% female) on a lacto-ovo-vegetarian diet and 40 children on a traditional omnivorous diet (45% male, 55% female) as controls. The lacto-ovo-vegetarian children did not consume meat, poultry, and fish, but ate eggs and dairy products and followed this diet since the introduction of complementary foods. The children remained under regular medical and nutritional supervision.

The exclusion criteria for the study were not being in the prepubertal period, infections of various etiologies and localizations, as well as intake of prescription medications and food supplements with anti-inflammatory and antioxidant properties, except for standard vitamin D3 supplementation, which amounted to 600–1000 IU/day [25].

### 2.2. Anthropometric Measurements and Dietary Assessment

The children’s height and weight were assessed using a standard stadiometer and electronic scale, respectively. Anthropometric measurements were taken using calibrated instruments. The same team examined all the study participants. Weight (kg) and height (m) were used to calculate BMI (body mass index). Body mass index was calculated as body weight (kg) divided by height squared (m^2^). BMI values were compared with BMI norms for age and sex according to the WHO criteria, thus obtaining a BMI z-score, which is a normalized relative weight indicator independent of age and sex [26,27]. Pubertal stage was assessed according to the Tanner’s criteria [28].

Dietary intakes were assessed using diet record methods. The parents of the studied children were advised by a nutritionist and asked to prepare a food diary for their children. Three dietary recalls (two weekdays and one weekend day) were performed to evaluate dietary habits. The average daily energy intake, i.e., percentage of energy from dietary protein, fat, and carbohydrates, as well as fiber and vitamin dietary intakes were assessed using the nutritional software program Dieta 5^®^ (National Food and Nutrition Institute, Warsaw), as described in more detail in the previous article [29].

### 2.3. Blood Sampling and Biochemical Analysis

For biochemical measurements, peripheral blood (3 mL) was taken in the morning after an overnight fast. Serum samples were obtained after centrifugation (2500× *g* at 4 °C for 10 min) and were used for C-reactive protein (CRP) determination. Serum levels of CRP were measured using immunoturbidimetric assay on the Cobas Integra auto-analyzer (Roche Diagnostics, Basel, Switzerland). Residual serum was stored in small portions at −70 °C for a maximum of four weeks until analyses of TOC, TAC, GSH, GSSG, and calprotectin were performed.

Concentrations of serum calprotectin were measured by enzyme-linked immunosorbent assay (ELISA) using specific antibodies with a high affinity to these proteins (CALPROLAB^TM^ Calprotectin (ALP) ELISA kit, CALPRO AS; Lysaker, Norway). The detection limit of this method was below 5.0 ng/mL and the intra- and inter-assay CVs were less than 5.0%.

Serum TOC and TAC values were evaluated by colorimetric assay based on the enzymatic reaction of peroxides and peroxidases according to Tatzber et al. [30] (Omnignostica Forschungs GmbH, Hoflein/Danube, Austria). The analytical sensitivity of TOC was 0.06 mmol/L, and the intra- and inter-assay CVs were 4.90% and 7.33%, respectively. The sensitivity of TAC was 0.08 mmol/L, and the intra- and inter-assay CVs were 5.00% and 6.92%, respectively. The oxidative stress index (OSI) was calculated from the ratio of TOC to TAC levels [31].

GSH and GSSG concentrations in serum were assessed using the human (GSH) ELISA kit and human (GSSG) ELISA kit (SunRed Biotechnology Company, Shanghai, China) based on sandwich enzyme-linked immunosorbent assay using two specific and high-affinity monoclonal antibodies. The intra- and inter-assay coefficients of variations (CVs) were less than 10% and 12% for GSH, and 8.0% and 11.0% for GSSG, respectively. The analytical sensitivity of the tests was 0.118 μmol/L for GSH and 0.045 μmol/L for GSSG, respectively. The GSH/GSSG ratio (R-index), which is regarded as an indicator of the redox state of the cell, was estimated.

### 2.4. Statistical Analysis

Statistical analysis was performed using SPSS software version 21. The Shapiro–Wilk test was used to check the normality of the data distribution. Parametric data were described as means and standard deviations (SDs) and were analyzed using Student’s *t* test. Non-parametric data were expressed as medians and interquartile ranges and were analyzed using the Mann–Whitney U test. The associations between biochemical parameters were calculated using Spearman’s rho. Differences were regarded as statistically significant at *p* < 0.05.

## 3. Results

Both groups of children were comparable in terms of age and anthropometric parameters (Table 1). 

An analysis of the children’s diets showed a similar daily energy intake in both groups of children (Table 2). Vegetarians had a significantly higher percentage of energy from carbohydrates, a lower percentage of energy from protein, and a similar percentage of energy from fat. Fiber intake was higher in vegetarians compared with omnivores. The intake of vitamin B_12_ was significantly lower in children on a vegetarian diet, while intakes of vitamins A, E, C, and D were similar in both groups.

The differences between serum concentrations of biochemical parameters in both groups of children included in the study are presented in Table 3. Children on a vegetarian diet had significantly lower median values of serum TOC, GSH, GSSG (*p* < 0.01) and significantly higher median values of TAC (*p* < 0.001) compared with the omnivores. OSI was significantly lower (*p* < 0.001) in vegetarian subjects than in controls, while R-index did not differ between both groups of children. CRP (within the reference value) and calprotectin median values did not significantly differ between both groups.

**Figure 1:**
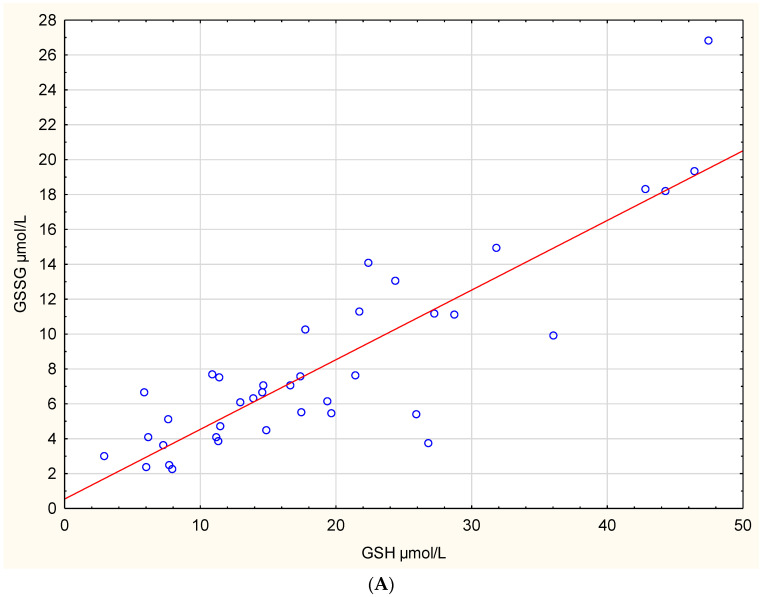

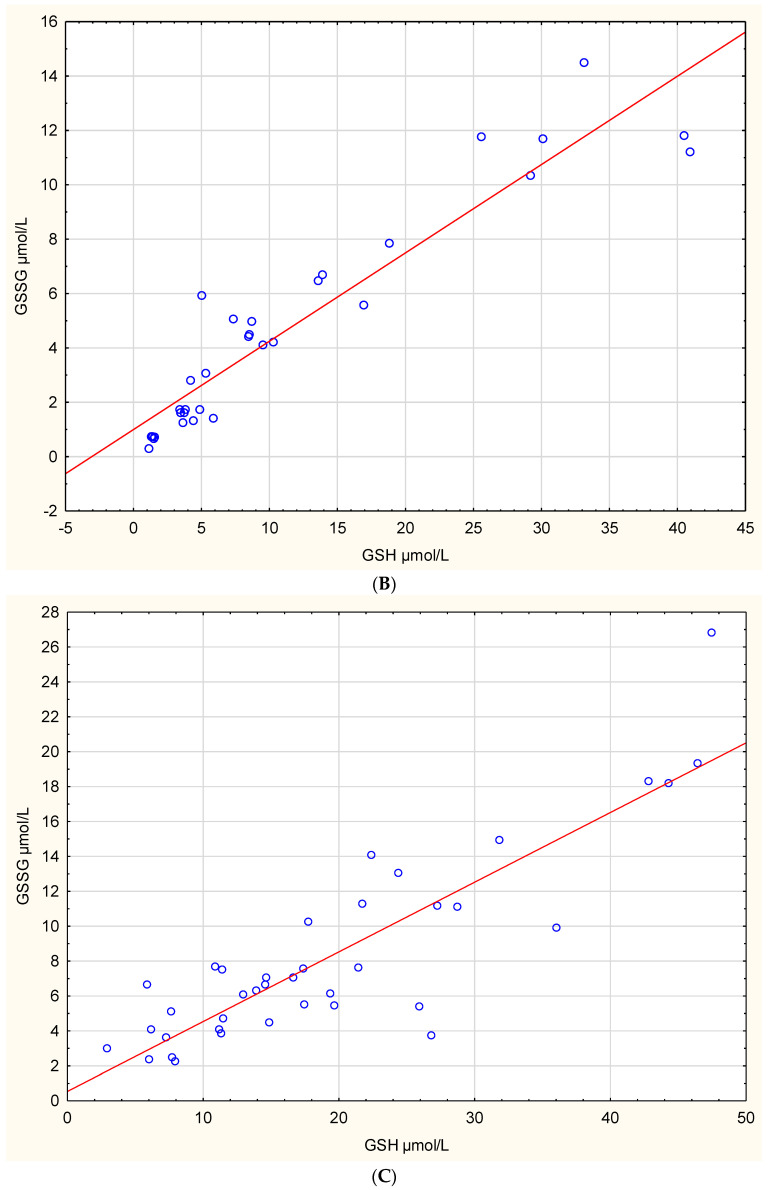
Correlations between serum GSH and GSSG in the whole group (**A**), vegetarian (**B**), and omnivorous (**C**) children.

Relations between antioxidant biochemical parameters were shown in Table 4 and Figure 1A–C. As expected, we observed a negative correlation (*p* < 0.05) between the levels of TOC and TAC in vegetarians and in the whole group of children. Moreover, in the whole group of children, TOC positively correlated with GSH (*p* < 0.05), GSSG (*p* = 0.05) and R-index (*p* < 0.05) (Table 4).

We also documented a strong significant positive correlation between levels of GSH and GSSG in the whole group (rho = 0.896, *p* < 0.001), as well as in vegetarian (rho = 0.933, *p* < 0.001) and omnivore (rho = 0.763, *p* < 0.001) groups (Figure 1A–C).

We revealed no correlation between the components of the diet and markers of inflammation. Similarly, the components of the diet did not correlate with TOC, TAC, OSI and GSH, GSSG, and R-index.

## 4. Discussion

Research results evaluating the concentrations of selected markers of both oxidation and reduction processes in adults on a vegetarian diet are ambiguous, and there are few studies of this type among children [32,33,34,35]. Thus, we were interested to know whether the oxidant-antioxidant balance (for the assessment of which we used the concentrations of selected oxidative stress markers such as TOC, TAC, GSH, GSSG and R-ratio, and OSI) is maintained in children on a vegetarian diet remaining under the constant medical and dietary care [36]. Such research seems to be important considering that vegetarian diets are an increasingly popular alternative to the traditional diet. Parents eating according to the vegetarian model of nutrition also feed their children similarly [37,38,39]. Among the 32 children on a vegetarian diet, 19 children had parents who were both vegetarians, while the remaining children had one vegetarian (the mother) and one non-vegetarian parent.

Research results indicate that a factor predisposing to the development of many diseases is oxidative stress, which may be caused by, among others, low-grade inflammation generating reactive oxygen species [40,41,42].

Some studies show that a vegetarian diet may affect inflammatory biomarker concentrations, thus reducing the risk of chronic diseases. An inflammatory marker that has not yet been evaluated in vegetarians is the complex S100A8 and S100A9 proteins called calprotectin. Calprotectin is contained mainly in the cytosol of neutrophils and is secreted during inflammation. The determination of calprotectin concentration in the stool is used for diagnosing intestinal inflammation, including inflammatory bowel disease. It is also useful to monitor the course of these diseases, i.e., response to treatment and the occurrence of exacerbations. The determination of its concentration in both serum and plasma is used in the diagnosis of many inflammatory diseases, including autoimmune diseases [43,44].

In our study, we found no differences in calprotectin concentrations between both groups of children. Among the biomarkers of inflammation, C-reactive protein appears to be associated with the vegetarian diet, and its levels were found to be lower in vegetarians compared with omnivores in some studies of adults [32,45,46].

In two studies conducted on children, one which concerned teenagers proved no significant differences in CRP concentrations among vegetarians compared with omnivores, which is similar to our study, while in the second study, which concerned prepubertal vegetarian children, lower concentrations of this marker were found. [29,47].

Body mass index, length of time on a vegetarian diet, and how balanced it is in terms of ingredients with anti-inflammatory and antioxidant properties have been considered as factors that may impact inflammatory marker concentrations in vegetarians.

The lower body weight observed by some authors in vegetarians compared with omnivores may be potentially associated with a lower percentage of body fat, which, as a hormonally active tissue, is responsible for the release of pro-inflammatory factors, e.g., some hormones, adhesion molecules, growth factors, and cytokines [48,49,50,51].

The nutritional status of the examined children was normal and the period of being on a vegetarian diet had been approximately six years. According to a meta-analysis by Haghighatdoost et al. [45], the period of being on such a diet for CRP to respond to the beneficial effects of a vegetarian lifestyle is two years. With regard to the relation between individual components of the diet and the concentration of inflammatory markers, including CRP, the research results are ambiguous. They point to both a beneficial effect of eating whole grain cereals, nuts, or legumes on CRP concentration, as well as to the lack of such a relationship. In the above analysis, either an inverse or a lack of a relationship between the consumption of fruit and vegetables and CRP concentration was found [45,52,53,54,55,56,57,58,59].

A vegetarian diet may be associated with a higher intake of vitamins, e.g., A, C, and E, and their higher concentrations in the serum [60,61,62,63]. However, not all studies confirm this. Some indicated no differences in the concentrations of such vitamins as A or E, and even found their lower concentrations in the serum of vegetarians, which has been explained by the poor bioavailability of those vitamins from plant foods, among others, and may be of particular concern for vegans [13,64,65,66,67,68]. In the studied groups of children on vegetarian and traditional diets, no significant differences were found in the content of vitamins A and E in the diet, but differences in the intake of vitamins C and B_12_ were observed [29].

In our study, only the intake of vitamin B_12_ was significantly lower in the vegetarian group. Furthermore, in this group, approximately 50% of the children did not achieve the level recommended by nutrition standards for this vitamin, whereas dietary intake of vitamin B_12_ for omnivorous children was sufficient. A lower intake of vitamin D and a higher intake than the recommendations of vitamins A, C, and E were found in the diets of children from both groups [69]. Some authors argue that vegetarian diets may adversely affect vitamin B_12_ status, causing vegetarians to be predisposed to diseases in which oxidative stress and inflammation are involved [70,71,72]. Misra et al. [73] showed that vitamin B_12_ deficiency was associated with increased Malondialdehyde (MDA, a product of lipid peroxidation) levels and decreased TAC and GSH concentrations in adults.

Glutathione, which plays an important role in antioxidant defense, also has a role in nutrient metabolism and the regulation of cellular events, including gene expression, protein synthesis, cell proliferation and apoptosis, signal transduction, cytokine production, and immune response [74]. Disturbances in glutathione homeostasis expressed by an imbalance in the GSH/GSSG ratio are increasingly recognized as a factor predisposing to oxidative stress, and in consequence, to the development of many diseases. The best documented observations of this kind concern neurodegenerative diseases (Parkinson’s disease, Alzheimer’s disease), diabetes and its related complications, cardiovascular diseases, and cancers [75,76].

During the reduction processes, GSH is oxidized to GSSG by glutathione peroxidase, while the regeneration of the active form of GSH occurs as a result of GSSG reduction by glutathione reductase [77]. Glutathione status in children on a vegetarian diet has not been systematically investigated so far. In a group of adult vegetarians, both lower and the same glutathione concentrations were found compared with omnivores. These studies most often concerned total glutathione or its reduced form and glutathione peroxidase activity [42,60,78,79].

In the present study, we measured the concentrations of both forms of this compound and determined the GSH/GSSG ratio, which is crucial for cell activity and survival. The positive relationship between the reduced and oxidized forms of glutathione that we found, both in the vegetarians and omnivores, may prove the efficient enzymatic regeneration process of this compound in the studied groups of children.

The lower GSH and GSSG concentrations in vegetarians may indicate lower GSH/GSSG ratio activity to neutralize emerging free radicals in response to lower total oxidative activity levels in this group compared with omnivores. The reduced concentrations of both forms of glutathione in vegetarians may also potentially be a result of a lower supply of sulfur amino acids, which are precursors of glutathione, in their diet. This is a possibility considering that, in the diet of vegetarians, the percentage of energy from protein was significantly lower than in omnivores, and sulfur amino acids, such as cysteine or methionine, are contained mainly in protein-rich foods such as meat and dairy. However, it should be borne in mind that cereal products, as well as legumes and nuts, included in the vegetarian diet are also a good source of sulfur amino acids. Therefore, in order to assess the status of these amino acids in the body, it would be necessary to determine their serum concentrations. It should also be remembered that the glutathione concentration could have also been affected by polymorphisms of GCLC and GCLM genes encoding the enzyme responsible for its synthesis, i.e., glutamate cysteine ligase (GCL), which we had not analyzed. It is worth noting that, despite significant differences in the concentrations of both forms of glutathione in the groups of children on vegetarian and omnivorous diets, the R-index did not differ significantly. This may indicate a balance of redox processes in both groups. The balance of oxidation and reduction processes in both groups of children may also be supported by the fact that, despite significantly lower TOC and higher TAC in the vegetarian group, the concentrations of these markers in both groups of children were within the normal range. According to the manufacturer’s reference data (instructions of LDN Labor Diagnostika NordGmbH & Co. KG, Nordhorn, Germany), the expected TOC value is <0.35 mmol/L, while TAC—indicating sufficient antioxidant capacity—is >1.3 mmol/L. Similarly, in the study of Drabko and Kowalczyk [80], the average total antioxidant status (TAS) was 1.3 mmol/L in healthy children. Our previous observations showed lower TAS levels in vegetarian children compared with omnivores (1.21 vs. 1.30 mmol/L; *p* < 0.001, respectively), but it was also within the physiological range in both groups of children [34].

This difference may be due to the fact that we originally studied a much smaller group of older children, not all of whom were on a vegetarian diet from birth.

In a group of adult vegetarians, the total plasma antioxidant activity assessed by FRAP (Ferric-reducing ability of plasma) and TAC tests was not significantly different from that which was observed in the group of omnivores. At the same time, the authors showed both higher and comparable concentrations of lipid and protein peroxidation products in the studied groups [32,79,81].

Vegetarian diets, if unbalanced, can lead to nutritional deficiencies, including anti-inflammatory and antioxidant properties, predisposing vegetarians to oxidative stress. Although in our study we did not find a relationship between the individual components of the diet and the assessed markers, the results indicate the important role played by doctors and dieticians in the care of children fed unconventionally in the context of maintaining the oxidant-antioxidant balance.

## 5. Strengths and Limitations

There are few studies assessing the oxidant-antioxidant balance in children following unconventional diets. To the best of our knowledge, we are the first to attempt to simultaneously assess the severity of both oxidation and reduction processes in children on a lacto-ovo-vegetarian diet. We are also the first to use markers such as TOC, TAC, GSH, and GSSG for this purpose and calculate the oxidative stress index (OSI) and the R-index from these.

The presented study has several potential limitations. We analyzed relatively small samples, which reduced the power of our results. However, our data are from two groups of prepubertal children who were similar in terms of age, weight, height, and BMI. Assessment of a broader panel of oxidative stress markers could be considered; however, the markers we assessed seem to be particularly useful for evaluating the efficiency of the oxidant-antioxidant mechanisms, and thus the predisposition to free radical complications.

The factors that may influence the severity of oxidative stress, including the development of potential oxidative stress-related complications, include not only the type of diet and its balance, but also its duration—as shown in studies on adults. Cho and Park [45] showed that a long-term vegetarian diet, i.e., for at least 15 years, had a positive effect on the total antioxidant status assessed on the basis of biological antioxidant potential (BAP) and endogenous antioxidant enzyme levels, such as superoxide dismutase, catalase, and glutathione peroxidase. In the studied group of children, the average duration of the vegetarian diet was only about six years, which could be considered another limitation. Nevertheless, this is the only study assessing the severity of oxidative stress in children on a lacto-ovo-vegetarian diet for so long.

Another limitation of our work may be the fact that we assessed the content of vitamins with antioxidant properties in the children’s diets, but we did not determine the concentrations of these vitamins in serum.

We consider this to be our pilot study. We plan to conduct this kind of observation while taking into account a broader panel of oxidative stress markers with a determination of serum antioxidant vitamin levels, not only in a larger group of children, but also in those following a vegetarian model of nutrition for a longer period of time. Taking into account that such children remain under the observation of our outpatient gastroenterology clinic from birth until adulthood, i.e., until the age of 18, it will be possible to conduct this type of study.

## 6. Conclusions

Our study results suggest that the vegetarian model of nutrition in children under regular medical and dietary care allows to maintain the oxidant-antioxidant balance in the serum of these children. Further studies with a larger number of subjects and with a longer follow-up period are needed to confirm our observations and to answer the question of whether a vegetarian diet will maintain the oxidant-antioxidant balance in these children in the future and whether it will influence the parameters of their somatic development and the risk of developing selected metabolic and cardiovascular diseases.

## Figures and Tables

**Table 1:** Anthropometric parameters in vegetarian and omnivorous children.

	Vegetarians (n = 32)	Omnivores (n = 40)	*p*
Age (years) *^+^*	6.6 ± 2.5	6.4 ± 2.4	0.725
Body weight (kg) *^++^*	22.7 (16.1–27.2)	22.1 (17.2–28.6)	0.371
Body height (cm) *^++^*	121.5 (104.5–133.5)	121.0 (104.5–132.7)	0.910
BMI (kg/m^2^) *^++^*	15.4 (14.6–16.1)	15.5 (15.1–18.1)	0.056
BMI z-score *^+^*	−0.18 ± 0.9	+0.19 ± 0.8	0.060

Data are presented as: ^+^ mean value and standard deviation (SD); ^++^ median value and interquartile ranges (Q1–Q3); BMI—body mass index; BMI z-score—a normalized relative weight indicator independent of age and sex; *p* < 0.05 was considered significant.

**Table 2:** Dietary intake of energy, macronutrients and vitamins in children on vegetarian and omnivore diets.

Diet	Vegetarians (n = 32)	Omnivores (n = 40)	*p*
Total energy (kcal)	1499.3 (1187.3–1723.4)	1558.4 (1239.5–1760.8)	0.330
Percentage of energy from protein (%)	11.6 (9.8–12.5)	13.2 (12.1–14.5)	0.010
Percentage of energy from fat (%)	28.9 (27.3–32.0)	31.3 (29.4–35.4)	0.079
Percentage of energy from carbohydrates (%)	56.7 (53.3–61.3)	52.9 (49.3–58.0)	0.005
Fiber (g/day)	18.5 (14.8–25.5)	14.2 (11.4–17.1)	0.012
Vitamin A (µg/day)	830.3 (534.5–1056.1)	748.7 (534.6–1009.6)	0.939
Vitamin E (mg/day)	9.6 (7.6–11.0)	9.1 (5.7–10.1)	0.277
Vitamin C (mg/day)	106.7 (51.4–135.7)	87.7 (59.9–110.2)	0.604
Vitamin D (µg/day)	1.4 (0.8–3.1)	1.4 (0.9–2.0)	0.781
Vitamin B_12_ (µg/day)	1.2 (0.9–1.5)	2.0 (1.8–2.3)	0.000

Data are presented as median values and interquartile ranges (Q1–Q3); *p* < 0.05 was considered significant.

**Table 3:** Differences in biochemical parameters between children on vegetarian and omnivorous diets.

Variables	Vegetarians (n = 32)	Omnivores (n = 40)	*p*
TOC (mmol/L)	0.06 (0.04–0.18)	0.16 (0.13–0.27)	0.001
TAC (mmol/L)	1.84 (1.57–2.50)	1.32 (1.12–1.64)	0.000
OSI	0.04 (0.02–0.10)	0.14 (0.08–0.22)	0.000
GSH (µmol/L)	6.61 (3.69–15.42)	17.03 (11.19–25.99)	0.001
GSSG (µmol/L)	4.15 (1.50–6.57)	6.63 (4.49–11.10)	0.002
R-index	2.25 (1.94–2.82)	2.36 (1.87 ± 2.94)	0.790
CRP (mg/L)	0.40 (0.30–0.70)	0.30 (0.10–0.60)	0.190
Calprotectin (ng/mL)	1002.5 (577.7–1541.7)	765.9 (580.7–992.9)	0.086

Data are presented as median values and interquartile ranges (Q1–Q3); *p* < 0.05 was considered significant. TOC—total oxidant capacity; TAC—total antioxidant capacity; OSI—oxidative stress index (TOC/TAC); GSH—reduced form of glutathione; GSSG—oxidized glutathione; R-index—GSH/GSSG ratio; CRP—C-reactive protein.

**Table 4:** Correlations between antioxidants parameters in vegetarian and omnivorous as well as in the whole group of children.

Biochemical Parameters	TOC		TAC	
	Rho	*p*	Rho	*p*
Lacto-ovo-vegetarians				
TOC	-	-	−0.445	0.011
TAC	−0.445	0.011	-	-
OSI	0.932	0.000	−0.718	0.000
GSH	−0.072	0.697	−0.270	0.136
GSSG	−0.048	0.795	−0.209	0.251
R-index	0.245	0.176	−0.290	0.108
Omnivores				
TOC	-	-	0.109	0.502
TAC	0.109	0.502	-	-
OSI	0.837	0.000	−0.354	0.025
GSH	0.250	0.130	0.292	0.075
GSSG	0.096	0.567	0.302	0.065
R-index	0.305	0.062	0.002	0.991
Whole group (lacto-ovo-vegetarians and omnivores)				
TOC	-	-	−0.288	0.014
TAC	−0.288	0.014	-	-
OSI	0.923	0.000	−0.583	0.000
GSH	0.301	0.011	−0.215	0.075
GSSG	0.235	0.050	−0.161	0.184
R-index	0.277	0.020	−0.094	0439

TOC—total oxidant capacity; TAC—total antioxidant capacity; OSI—oxidative stress index (TOC/TAC); GSH—reduced form of glutathione; GSSG—oxidized glutathione; R-index—GSH/GSSG ratio.

## Data Availability

The data is contained within the article.
